# Epithelial-mesenchymal transition-related gene expression as a new prognostic marker for neuroblastoma

**DOI:** 10.3892/ijo.2012.1684

**Published:** 2012-11-06

**Authors:** MEGUMI NOZATO, SETSUKO KANEKO, AKIRA NAKAGAWARA, HIROAKI KOMURO

**Affiliations:** 1 Department of Pediatric Surgery, Faculty of Medicine, University of Tsukuba, Tsukuba;; 2 Chiba Cancer Center Research Institute, Chiba;; 3 Department of Pediatric Surgery, Graduate School of Medicine, University of Tokyo, Tokyo, Japan

**Keywords:** neuroblastoma, epithelial-mesenchymal transition, invasive ability, keratin19, ERBB3 (HER3)

## Abstract

Neuroblastoma (NB) is a highly metastatic tumor in children. The epithelial-mesenchymal transition (EMT) is an important mechanism for both the initiation of tumor invasion and subsequent metastasis. This study investigated the role of EMT in the progression of NB. Using EMT assays on samples from 11 tumors, we identified 14 genes that were either differentially expressed between tumors of different stages or highly upregulated in NB. Quantitative RT-PCR of these genes was conducted in 96 NB tumors and their expression levels were compared between stages and between tumors with the presence and absence of *MYCN* amplification. The association of survival rate with differential gene expression was investigated. Expression of *KRT19* was significantly decreased in stage 3 or 4 NB as well as stage 4S NB compared with stage 1 or 2 NB. Expression levels of *KRT19* and *ERBB3* were significantly low, and expression levels of *TWST1* and *TCF3* were high in *MYCN*-amplified NB. The patients with low expression of *KRT19* or *ERBB3* showed significantly worse overall survival. Furthermore, the correlation between high invasive ability and low expression of *KRT19* and *ERBB3* was suggested *in vitro* using six NB cell lines. The authors conclude that downregulation of *KRT19* is highly associated with tumor progression in NB and metastasis in localized primary NB and that low expression of *ERBB3* is also associated with progression of NB.

## Introduction

Neuroblastoma (NB) is one of the most common pediatric solid tumors, accounting for 15% of all pediatric cancer deaths. It originates from the sympathoadrenal lineage derived from the neural crest. The clinical behavior is markedly heterogeneous (1–3). Most tumors tend to grow aggressively and often have a fatal outcome, but some tumors are favorable and show spontaneous differentiation or regression. The stage of the tumor at diagnosis, the age of the patient and the presence or absence of *MYCN* amplification are the basic parameters used for risk stratification to determine the management and treatment of this disease. Recent progress in chemotherapy has dramatically increased the survival rates of many pediatric cancers; however, advanced stage NB with metastasis, especially those with genomic amplification of the *MYCN* oncogene, are frequently resistant to any therapy and the outcome for patients is still very poor (1–3). Therefore, it is important to know the mechanism of metastasis in NB in order to improve the treatment results.

The epithelial-mesenchymal transition (EMT) is a series of events during which epithelial cells lose many of their epithelial characteristics and take on properties typical of mesenchymal cells. EMT has an important role in the development of many tissues during embryogenesis and similar cell changes are recapitulated during pathological processes, such as fibrosis and cancer. Numerous observations support the idea that EMT has a central role in tumor progression and metastasis (4–7). Cancer cells acquire mesenchymal gene expression patterns and properties, resulting in reduced cell-cell adhesion and the activation of proteolysis and motility. These activities promote tumor invasion and metastasis. EMT is important in the progression of tumor cells acquiring a more invasive, metastatic capacity. In this study, we investigated the role of EMT in the progression of NB in terms of invasiveness and metastasis.

## Materials and methods

### Tumor samples

Ninety-six primary NBs were obtained from the Department of Pediatric Surgery, University of Tsukuba, and the Division of Biochemistry, Chiba Cancer Center Research Institute, Japan. Patients were aged between 0 months and 18 years at diagnosis (median 16 months). The clinical characteristics of the 96 NBs are shown in Table I.

### Cell lines

Six NB cell lines (SK-N-AS, SK-N-DZ, SK-N-SH, GOTO, GANB and TGW) were used for invasion assays. SK-N-SH, SK-N-DZ and SK-N-AS were kindly provided by Toru Sugimoto, Kyoto Prefectural Medical University. TGW and GANB were provided by Chiba Cancer Center. GOTO was purchased from American Type Culture Collection (Manassas, VA, USA). These were maintained in Daigo’s medium supplemented with 10% fetal bovine serum (BioWest, Nuaille, France) at 37°C in a humidified 5% CO_2_ atmosphere.

### RNA extraction and cDNA transcription

Total-RNA was prepared from frozen tumor tissue by the guanidine isothiocyanate-phenol method using Isogen (Wako Junyaku Kogyo, Tokyo, Japan) according to the manufacturer’s instructions. One microgram of each RNA was reverse transcribed to cDNA with random hexamer primers and transcriptor reverse transcriptase using the Transcriptor First Strand cDNA Synthesis Kit (Roche, USA).

### EMT assay

To examine the expression levels of the EMT-related genes, we used an RT^2^ Profiler PCR Array for human EMT (SA Biosciences) consisting of quantitative RT-PCR of 84 EMT-related genes. This array coated 96-well microtiter plates and was performed using an ABI Prism 7700 Sequence Detection System (Applied Biosystems, Foster City, CA, USA) according to the following program: 95°C for 10 min, 43 cycles at 95°C for 15 sec and then 60°C for 1 min.

### Real-time quantitative RT-PCR

The expression levels of cardesmon 1 (*CALD1*), epidermal growth factor (*EGFR*), desmoplakin (*DSP*), secreted protein acidic and rich in cysteine (*SPARC*), zinc finger E-box-binding homeobox 1 (*ZEB1*), zinc finger E-box-binding homeobox 2 (*ZEB2*), fibronectin 1 (*FN1*), vimentin (*VIM*), keratin 19 (*KRT19*), erythroblastic leukemia viral oncogene homolog (*ERBB3*), regulator of G-protein signaling 2 (*RGS2*), transcription factor 3 (*TCF3*) and *TWIST1* were measured by the ABI Prism 7700 Sequence Detection System (Applied Biosystems) using Universal ProbeLibrary (UPL)-based real-time quantitative RT-PCR (Roche Diagnostics). UPL is based on only 165 short hydrolysis probes of just 8–9 nucleotides, each of which is labeled at the 5′ end with FAM and at the 3′ end with a dark quencher dye. Human *ACTNB (*β-actin) was used as an internal control gene. The specific primers used are shown in Table II. The UPL probes used were nos. 52, 69, 78, 7, 77, 3, 68, 13, 33, 71, 37, 61, 35, 6 and 64 in UPL for *CALD1, EGFR, DSP, SNAIL2, SPARC, ZEB1, ZEB2, VIM, FN1, KRT19, ERBB3, RGS2, TCF3, TWIST1* and human *ACTNB*, respectively. Each experiment was carried out with each sample in triplicate and repeated twice. The thermal cycling conditions were as follows: 50°C for 2 min, 95°C for 10 min, 40 cycles at 95°C for 15 sec and then 60°C for 1 min. Data from real-time PCR were calculated using the ΔΔC_t_ method as previously described (8).

### Matrigel invasion assay

The invasive ability of NB cell lines was measured using BD Falcon cell culture inserts with an 8-*μ*m pore size PET membrane and 24-well BD BioCoat Matrigel Invasion Chambers (BD Biosciences, Bedford, MA, USA) according to the manufacturer’s instructions. NB cell suspensions were adjusted to 1.0×10^5^ cells per well on Matrigel invasion chamber plates and non-matrigel coat invasion chamber (control inserts) and cultured in routine medium in the absence or presence of FBS. After incubation at 37°C under 5% CO_2_ for 72 h, the cells that had invaded the chamber and migrated to the lower surface were stained with Diff-Quik (Sysmex, Kobe, Japan) and manually counted under a microscope. The invading cells were stained and counted in 5 random fields at ×100 magnification. The mean number of counted cells was defined as the invasive ability. Each experiment was repeated 3 times.

### Statistical analysis

Survival analysis was performed according to the Kaplan-Meier method and the log-rank test. Relative mRNA expression levels were expressed as the mean ± SD. Student’s or Welch’s t-tests were used to assess the significance of differences between the groups. A p-value of <0.01 was considered statistically significant. This study was approved by the institutional ethics committee for human genome research of the University of Tsukuba (no. 211).

## Results

### Analysis of EMT-related gene expression in 11 NB tumors using EMT assay

Eleven NB tumors in various stages (Table III) were analyzed by EMT multiple gene profiling microarray. The expressions of 84 EMT-related genes were compared among the 11 tumors using the EMT assay. Seven genes (*CALD1, EGFR, DSP, SNAIL2, SPARC, ZEB1* and *ZEB2*) were found to be differentially expressed between NBs with low stages (stages 1 or 2) and those with high stages (stages 3 or 4). Five genes (*KRT19*, *ERBB3*, *RGS2*, *TCF3* and *TWIST1*) were found to be differentially expressed between *MYCN*-amplified and *MYCN*-non-amplified tumors. These genes and two others highly expressed in NB tumors (*VIM* and *FN1*) were further analyzed in 96 tumors using quantitative PCR.

### Correlation of EMT-related gene expression between low and high tumor stages

**Figure 1.**
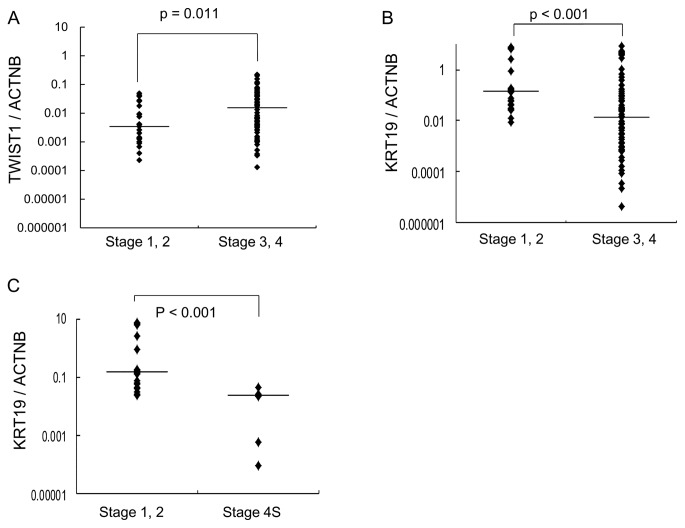
(A and B) Differential expression of EMT-related genes between low-stage NB (stage 1 or 2) and high-stage NB (stage 3 or 4). (A) *TWIST1* was more highly expressed in high-stage NB (p=0.011). (B) Significantly low expression of *KRT19* was associated with high stages of NB. (C) Differential expression of *KRT19* between localized stage 1 or 2 NB and metastatic localized stage 4S NB. *KRT19* was downregulated in metastatic stage 4S NB compared with localized stage 1 or 2 NB.

The expression levels of *ERBB3, RGS2, TCF3, CALD1, EGFR, DSP, SNAIL2, SPARC, ZEB1, ZEB2, VIM* and *FN1* did not show any significant differences between low- and high-stage NB. In contrast, low expression of *KRT19* was significantly associated with high stages of NB (Fig. 1B). *TWIST1* was found to be highly expressed in stage 3 or 4 NB (p=0.011) (Fig. 1A).

### Correlation of EMT-related gene expression with metastasis in localized primary tumors

Expression of these EMT-related genes was compared between stage 1 or 2 localized NB and stage 4S NB. Expression of *KRT19* was significantly lower in stage 4S NB, which develops metastasis in localized primary NB (Fig. 1C).

### Correlation of EMT-related gene expression with MYCN amplification

**Figure 2.**
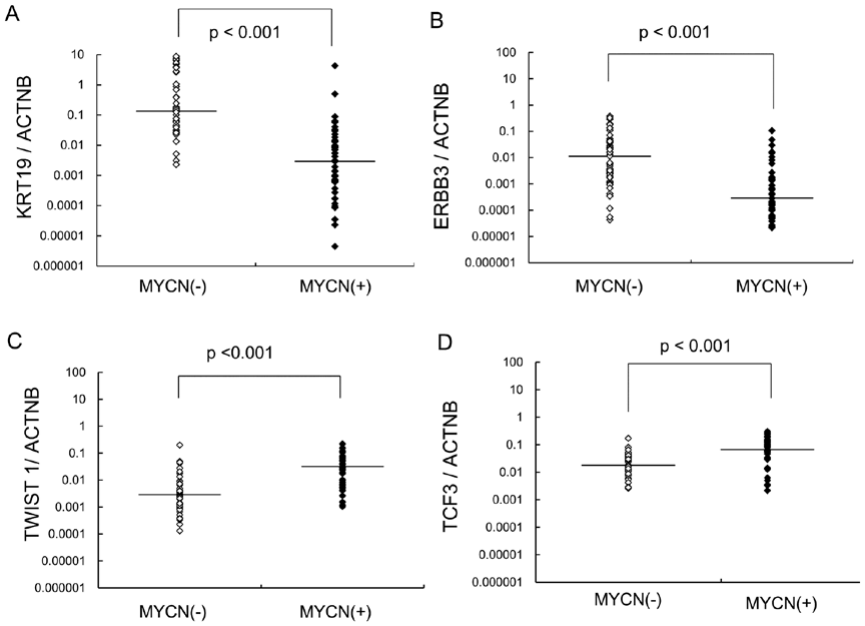
Differential expression of EMT-related genes between *MYCN*-amplified and *MYCN*-unamplified NB. (A and B) Expression of *KRT19* and *ERBB3* was significantly decreased in *MYCN*-amplified NB, (C and D) while expression of *TWIST1* and *TCF3* was significantly increased in *MYCN*-amplified NB. *MYCN*(+), *MYCN* amplification; *MYCN*(−), *MYCN* non-amplification.

Expression of these EMT-related genes was compared between NB with and without *MYCN* amplification. Expression of *KRT19* and *ERBB3* was significantly decreased in NB with *MYCN* amplification, while *TCF3* and *TWIST1* expression were increased (Fig. 2). *MYCN*-amplified NB showed significantly lower expression of *KRT19* and *ERBB3* compared with *MYCN*-unamplified NB.

### Overall survival rates for tumors with VIM, FN1, KRT19, ERBB3, TCF3 and TWIST1 gene misregulation

**Figure 3.**
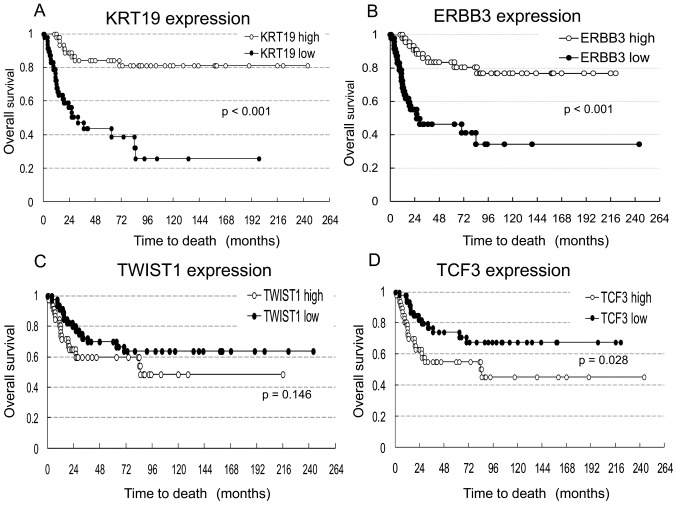
Kaplan-Meier survival analysis of 94 NB patients, stratified by their status of *KRT19*, *ERBB3*, *TWIST1* and *TCF3* gene expression. (A and B) The patients with low expression of *KRT19* or *ERBB3* in tumor tissues had significantly inferior survival compared with those with high expression. (C and D) No significant difference was observed between patients with high and low expression of *TWIST1* and *TCF3* genes.

Survival analysis was conducted in 94 NB tumors excluding 2 in which NB was not the cause of death. These NBs were divided into two groups: high expressers (47 NBs) and low expressers (47 NBs) of 6 genes (*VIM, FN1, KRT19, ERBB3, TCF3* and *TWIST1*). The median of log-transformed mRNA expression level was used as the cut-off value. Kaplan-Meier survival curves were compared for each gene between tumors with high and low expression (Fig. 3). The graph shows a trend toward increased survival for NB patients with increased *KRT19* or *ERBB3* expression. Expression levels of the other genes (*VIM, FN1*, *TCF3* and *TWIST1*) were not associated with patient survival.

### The correlation of low KRT19 and ERBB3 expression with invasive ability in NB cell lines

**Figure 4.**
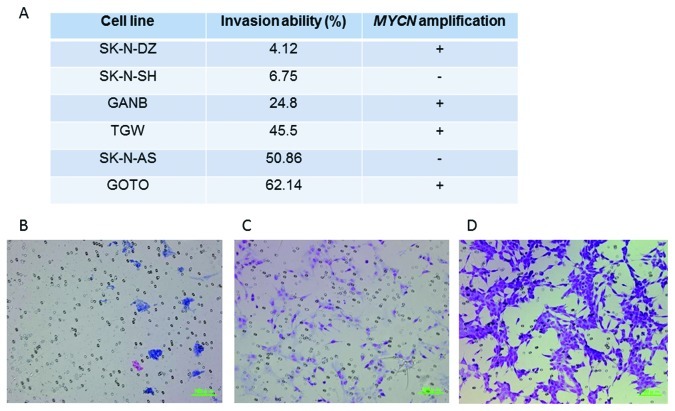
Results of Matrigel invasion assay in 6 NB cell lines. (A) Two cell lines (SK-N-DZ and SK-N-SH) showed low invasive abilities, while the other four cell lines (GANB, TGW, SK-N-AS and GOTO) showed high invasive abilities. (B) SK-N-DZ, (C) SK-N-AS and (D) GOTO cells are shown to be capable of migrating through the matrigel.

**Figure 5.**
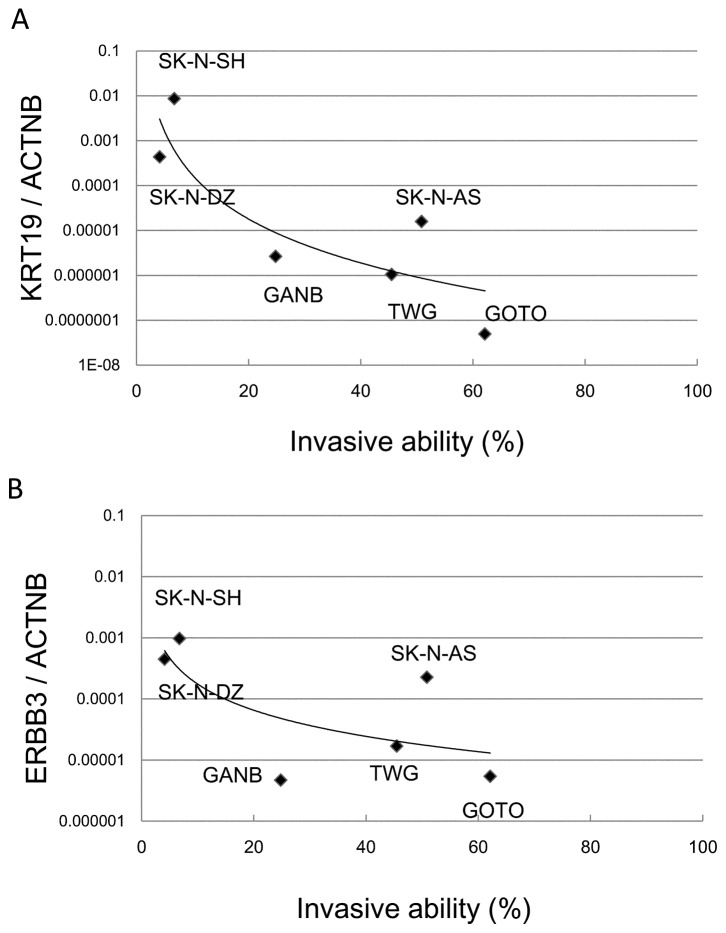
Effects of *ERBB3* and *KRT19* mRNA expression on *in vitro* tumor cell invasiveness. Note the markedly decreased invasive abilities in the cell lines with high expression of (A) *KRT19* and (B) *ERBB3* in comparison to those with low expression.

A Matrigel invasion assay demonstrated that two cell lines (SK-N-SH and SK-N-DZ) showed significantly reduced invasive ability (6.75 and 4.12%, respectively) while the 4 other cell lines (GANB, TGW, SK-N-AS and GOTO) showed high invasive abilities (24.8, 45.5, 50.86 and 62.1%, respectively) (Fig. 4). The correlation of *KRT19* and *ERBB3* expression with invasive abilities was investigated in the cell lines. The decreased expression of *KRT19* or *ERBB3* was highly correlated with invasiveness in NB cell lines (Fig. 5A and B). SK-N-DZ, with high expression of *KRT19* and *ERBB3* and *MYCN* amplification, had low invasive ability; while SK-N-AS, with low expression of *KRT19* and *MYCN* non-amplification, showed high invasive ability.

## Discussion

In this study, four EMT-related genes (*KRT19*, *ERBB3*, *TWIST1* and *TCF3*) were found to be differentially expressed. Expression of *KRT19* was significantly decreased in high-stage NB compared to low-stage NB (Fig. 1B). Downregulation of *KRT19* gene expression was highly associated with tumor progression in NB. Furthermore, expression of *KRT19* was markedly decreased in NB with *MYCN* amplification. Decreased expression of *KRT19* was found to be significantly associated with poor prognosis (Fig. 3). Interestingly, expression of *KRT19* was significantly decreased in metastatic favorable stage 4S NB compared to localized favorable stage 1 or 2 NB (Fig. 1C). These findings show that decreased expression of *KRT19* is strongly associated with the promotion of metastasis in favorable NB. Keratin is an epithelial marker, and downregulation of keratins is associated with EMT. Dysregulation of keratin expression has long been recognized as a feature of epithelial tumor progression (9). A recent report also demonstrated that expression of *KRT19* mRNA was significantly lower in tumors from patients that have died from NB compared with patients with no evidence of disease, and that low methylation of *KRT19* was associated with a favorable outcome (10). Supporting this, our results demonstrated that low expression of *KRT19* was significantly associated with high tumor stages, *MYCN* amplification and an unfavorable outcome in NB.

*TWIST1* is a key regulator of embryogenesis and is also known to be an EMT inducer. *TWIST1* belongs to the basic helix-loop-helix (bHLH) transcription factor family and promotes EMT by repressing the expression of E-cadherin, which leads to disassembly of adherens junctions and increased migratory potential (11). The link between *TWIST1* expression and metastasis is clear and well established (11,12). *TWIST1* is known to be overexpressed in *MYCN*-amplified NB tumors and cell lines and is responsible for the inhibition of the ARF/p53 pathway involved in the MYC-dependent apoptotic response. The cooperation of *TWIST1* and *MYCN* is thought to cause cell transformation and malignant outgrowth (13,14). In this study, *TWIST1* was highly expressed in *MYCN*-amplified NB as well as in high-stage NB. However, the survival rates between patients with low and high expression of *TWIST1* were not significantly different (p= 0.146), so its utility as a mesenchymal marker may be limited.

*TCF3* (E12/E47) is a basic bHLH transcription factor. A previous study implicated *TCF3* as a repressor of E-cadherin promoter activity and demonstrated its involvement in the acquisition and maintenance of the mesenchymal phenotype (15). In this study, high expression of *TCF3* was associated with *MYCN* amplification in NB. Survival rates were not significantly different between patients with high and low expression of *TCF3*.

*ERBB3* is a member of the epidermal growth factor receptor (EGFR) family, which is composed of *EGFR, ERBB2* (*HER2*), *ERBB3* (*HER3*) and *ERBB4* (*HER4*). Although *ERBB3* lacks an active tyrosine kinase domain, it can heterodimerize with other *ERBB* receptors. Heterodimerization leads to the activation of pathways which lead to cell proliferation or differentiation. The role of EGFR in the proliferation of NB, and the utility of its inhibitors in the treatment of NB, have all been well documented; however, the data remain somewhat contradictory (16,17), as other reports have demonstrated that exposure to EGF can induce apoptosis in NB through the *ERBB2* and *ERBB3* receptors (18–20). Richards *et al* reported that non-EGFR ERBB family members (*ERBB2*, *ERBB3* and *ERBB4*) contributed to NB growth and survival, and that pan-ERBB inhibition, rather than an EGFR specific inhibitor, represents a potential therapeutic target (21). These findings suggest that *ERBB2*, *ERBB3* and *ERBB4* play a significant role in tumor progression of NB, but Gambini *et al* reported that expression of *ERBB2* was not related to tumor progression of NB (22). Although a recent immunohistological study suggested the significance of EGFR family expression as a prognostic factor in NB, showing that *EGFR* and *HER2* expression is found in favorable NB and high expression of *HER4* is found in metastatic NB, the role of HER family members in NB remains interrelated and complex (23). In our study, decreased expression of *ERBB3* was also correlated with *MYCN*-amplified NB and poor survival rate. Several lines of evidence that provide support for the pivotal role of *ERBB3* in human carcinogenesis have emerged in recent years (24). High expression of *ERBB3* in certain human cancers led early to the suggestion that it could be a therapeutic target (25–28), but in some cancer cells the mesenchymal phenotype was found to lose *ERBB3* expression and show resistance to EGFR inhibitors. Epithelial phenotype, however, maintained *ERBB3* expression (29,30). The EMT might decrease the cellular dependency upon EGF signaling by kinase switching; mesenchymal cells might acquire alternative survival signals, thus becoming resistant to EGFR inhibitors (30). Downregulation of *ERBB3* in NB might suggest similar kinase switching during the EMT followed by tumor survival with the loss of EGF dependency.

Next, we investigated the invasive abilities of six NB cell lines using a Matrigel invasion assay to confirm the association of tumor invasiveness with expression of *KRT19* and *ERBB3*. While SK-N-DZ and SK-N-SH cell lines had a low invasive ability (4.12 and 6.75%, respectively), the other cell lines showed a high invasive ability (24.8–62.14%) (Fig. 4). Both cell lines with a low invasive ability had low expression of *KRT19* and *ERBB3* compared with the other cell lines (Fig. 5A and B). Interestingly, SK-N-DZ showed a low invasive ability as expected from high expression levels of *KRT19* and *ERBB3*, although its *MYCN* amplification should give it a high invasive ability. Thus, although *MYCN* gene amplification is the most powerful prognostic factor in NB, the expression levels of *KRT19* or *ERBB3* might become another promising prognostic marker.

## Figures and Tables

**Table I.** Tumor stages and MYCN amplification of 96 neuroblastomas.

	Stage 1, 2	Stage 4S	Stage 3	Stage 4	Total
MYCN					
Unamplified	22	4	10	15	51
Amplified	2	2	11	30	45
Total	24	6	21	45	96

**Table II.** Sequences of the primers used for PCR.

Gene	Forward primer	Reverse primer
*CALD1*	5′-GAGCGTCGCAGAGAACTTAGA-3′	5′-TCCTCTGGTAGGCGATTCTTT-3′
*EGFR*	5′-GCCTTGACTGAGGACAGCA-3′	5′-TTTGGGAACGGACTGGTTTA-3′
*DSP*	5′-CTTTGCGCCAATTCAATTAAG-3′	5′-CCAGTCCTGAGGTGTATGAGG-3′
*SNAIL2*	5′-TGGTTGCTTCAAGGACACAT-3′	5′-GTTGCAGTGAGGGCAAGAA-3′
*SPARC*	5′-GTGCAGAGGAAACCGAAGAG-3′	5′-TGTTTGCAGTGGTGGTTCTG-3′
*ZEB1*	5′-GGGAGGAGCAGTGAAAGAGA-3′	5′-TTTCTTGCCCTTCCTTTCTG-3′
*ZEB2*	5′-AAGCCAGGGACAGATCAGC-3′	5′-CCACACTCTGTGCATTTGAACT-3′
*VIM*	5′-TACAGGAAGCTGCTGGAAGG-3′	5′-ACCAGAGGGAGTGAATCCAG-3′
*FN1*	5′-GGAAAGTGTCCCTATCTCTGATACC-3′	5′-AATGTTGGTGAATCGCAGGT-3′
*KRT19*	5′-GCCACTACTACACGACCATCC-3′	5′-CAAACTTGGTTCGGAAGTCAT-3′
*ERBB3*	5′-CTGATCACCGGCCTCAAT-3′	5′-GGAAGACATTGAGCTTCTCTGG-3′
*RGS2*	5′-GAAAAGGAAGCTCCAAAAGAGA-3′	5′-TTCTGGGCAGTTGTAAAGCA-3′
*TCF3*	5′-CTCGGTCATCCTGAACTTGG-3′	5′-TCTCCAACCACACCTGACAC-3′
*TWIST1*	5′-AAGGCATCACTATGGACTTTCTCT-3′	5′-GCCAGTTTGATCCCAGTATTTT-3′
*ACTNB*	5′-CCAACCGCGAGAAGATGA-3′	5′-CCAGAGGCGTACAGGGATAG-3′

**Table III.** Characteristics of 11 neuroblastomas used in EMT assay.

	Stage 1, 2	Stage 4S	Stage 3, 4	Total
MYCN				
Unamplified	1	1	1	3
Amplified	3	2	3	8
Total	4	3	4	11

## References

[1] Brodeur GM (2003). Neuroblastoma: biological insights into a clinical enigma. Nat Rev Cancer.

[2] Maris JM (2010). Recent advances in neuroblastoma. N Engl J Med.

[3] Maris JM (2005). The biologic basis for neuroblastoma heterogeneity and risk stratification. Curr Opin Pediatr.

[4] Thiery JP, Sleeman JP (2006). Complex networks orchestrate epithelial-mesenchymal transitions. Nat Rev Mol Cell Biol.

[5] Yang J, Weinberg RA (2008). Epithelial-mesenchymal transition: at the crossroads of development and tumor metastasis. Dev Cell.

[6] Lee JM, Dedhar S, Kalluri R, Thompson EW (2006). The epithelial-mesenchymal transition: new insights in signaling, development, and disease. J Cell Biol.

[7] Thiery JP (2002). Epithelial-mesenchymal transitions in tumour progression. Nat Rev Cancer.

[8] Livak KJ, Schmittgen TD (2001). Analysis of relative gene expression data using real-time quantitative PCR and the 2^−ΔΔCt^ method. Methods.

[9] Moll R, Franke WW, Schiller DL, Geiger B, Krepler R (1982). The catalog of human cytokeratins: patterns of expression in normal epithelia, tumors and cultured cells. Cell.

[10] Carén H, Djos A, Nethander M, Sjöberg RM, Kogner P, Enström C, Nilsson S, Martinsson T (2011). Identification of epigenetically regulated genes that predict patient outcome in neuroblastoma. BMC Cancer.

[11] Yang J, Mani SA, Donaher JL, Ramaswamy S, Itzykson RA, Come C, Savagner P, Gitelman I, Richardson A, Weinberg RA (2004). Twist, a master regulator of morphogenesis, plays an essential role in tumor metastasis. Cell.

[12] Karreth F, Tuveson DA (2004). Twist induces an epithelial-mesenchymal transition to facilitate tumor metastasis. Cancer Biol Ther.

[13] Valsesia-Wittmann S, Magdeleine M, Dupasquier S, Garin E, Jallas AC, Combaret V, Krause A, Leissner P, Puisieux A (2004). Oncogenic cooperation between H-Twist and N-Myc overrides failsafe programs in cancer cells. Cancer Cell.

[14] Puisieux A, Valsesia-Wittmann S, Ansieau S (2006). A twist for survival and cancer progression. Br J Cancer.

[15] Perez-Moreno MA, Locascio A, Rodrigo I, Dhondt G, Portillo F, Nieto MA, Cano A (2001). A new role for E12/E47 in the repression of E-cadherin expression and epithelial-mesenchymal transitions. J Biol Chem.

[16] Ho R, Minturn JE, Hishiki T, Zhao H, Wang Q, Cnaan A, Maris J, Evans AE, Brodeur GM (2005). Proliferation of human neuroblastomas mediated by the epidermal growth factor receptor. Cancer Res.

[17] Tamura S, Hosoi H, Kuwahara Y, Kikuchi K, Otabe O, Izumi M, Tsuchiya K, Iehara T, Gotoh T, Sugimoto T (2007). Induction of apoptosis by an inhibitor of EGFR in neuroblastoma cells. Biochem Biophys Res Commun.

[18] Chiu B, Mirkin B, Madonna MB (2007). Epidermal growth factor can induce apoptosis in neuroblastoma. J Pediatr Surg.

[19] Chiu B, Mirkin B, Madonna MB (2006). Mitogenic and apoptotic actions of epidermal growth factor on neuroblastoma cells are concentration-dependent. J Surg Res.

[20] Chiu B, Mirkin B, Madonna MB (2007). Novel action of epidermal growth factor on caspase 3 and its potential as a chemotherapeutic adjunct for neuroblastoma. J Pediatr Surg.

[21] Richards KN, Zweidler-McKay PA, Van Roy N, Speleman F, Trevino J, Zage PE, Hughes DP (2010). Signaling of ERBB receptor tyrosine kinases promotes neuroblastoma growth in vitro and in vivo. Cancer.

[22] Gambini C, Sementa AR, Boni L, Marino CE, Croce M, Negri F, Pistoia V, Ferrini S, Corrias MV (2003). Expression of HER2/neu is uncommon in human neuroblastic tumors and is unrelated to tumor progression. Cancer Immunol Immunother.

[23] Izycka-Swieszewska E, Wozniak A, Drozynska E, Kot J, Grajkowska W, Klepacka T, Perek D, Koltan S, Bien E, Limon J (2011). Expression and significance of HER family receptors in neuroblastic tumors. Clin Exp Metastasis.

[24] Sithanandam G, Anderson LM (2008). The ERBB3 receptor in cancer and cancer gene therapy. Cancer Gene Therapy.

[25] Gullick WJ (1996). The c-erbB3/HER3 receptor in human cancer. Cancer Surv.

[26] Travis A, Pinder SE, Robertson JF, Bell JA, Wencyk P, Gullick WJ, Nicholson RI, Poller DN, Blamey RW, Elston CW, Ellis IO (1996). C-erbB-3 in human breast carcinoma: expression and relation to prognosis and established prognostic indicators. Br J Cancer.

[27] Sergina NV, Rausch M, Wang D, Blair J, Hann B, Shokat KM, Moasser MM (2007). Escape from HER-family tyrosine kinase inhibitor therapy by the kinase-inactive HER3. Nature.

[28] Baselga J, Swain SM (2009). Novel anticancer targets: revisiting ERBB2 and discovering ERBB3. Nat Rev Cancer.

[29] Fuchs BC, Fujii T, Dorfman JD, Goodwin JM, Zhu AX, Lanuti M, Tanabe KK (2008). Epithelial-to-mesenchymal transition and integrin-linked kinase mediate sensitivity to epidermal growth factor receptor inhibition in human hepatoma cells. Cancer Res.

[30] Thomson S, Petti F, Sujka-Kwok I, Epstein D, Haley JD (2008). Kinase switching in mesenchymal-like non-small cell lung cancer lines contributes to EGFR inhibitor resistance through pathway redundancy. Clin Exp Metastasis.

